# Salivary Gland Derived BDNF Overexpression in Mice Exerts an Anxiolytic Effect

**DOI:** 10.3390/ijms18091902

**Published:** 2017-09-05

**Authors:** Juri Saruta, Masahiro To, Masahiro Sugimoto, Yuko Yamamoto, Tomoko Shimizu, Yusuke Nakagawa, Hiroko Inoue, Ichiro Saito, Keiichi Tsukinoki

**Affiliations:** 1 Department of Oral Science, Graduate School of Dentistry, Kanagawa Dental University, 82 Inaoka, Yokosuka, Kanagawa 238-8580, Japan; saruta@kdu.ac.jp (J.S.); m.tou@kdu.ac.jp (M.T.); msugi@sfc.keio.ac.jp (M.S.); yusuke_2828@yahoo.co.jp (Y.N.); 2 Institute for Advanced Biosciences, Keio University, Tsuruoka, Yamagata 708-0813, Japan; 3 Health Promotion and Preemptive Medicine, Research and Development Center for Minimally Invasive Therapies, Tokyo Medical University, Sinjuku, Tokyo 100-0001, Japan; 4 School of Dental Hygiene, Department of Junior College, Kanagawa Dental University, Yokosuka, Kanagawa 238-8580, Japan; yuko@yd6.so-net.ne.jp; 5 Department of Highly Advanced Stomatology, Yokohama, Kanagawa Dental University, Graduate School of Dentistry, Yokohama, Kanagawa 221-0835, Japan; shimizu@kdu.ac.jp; 6 Department of Pathology, Tsurumi University School of Dental Medicine, Yokohama, Kanagawa 238-8580, Japan; inoue-h@nichiyaku.ac.jp (H.I.); saito-i@tsurumi-u.ac.jp (I.S.); 7 Department of Pharmaceutical Sciences, Nihon Pharmaceutical University, Kitaadachi, Saitama 335-0021, Japan

**Keywords:** BDNF, transgenic mouse, hippocampus, saliva, salivary gland, TrkB, anxiolytic

## Abstract

Brain-derived neurotrophic factor (BDNF) is abundant in the hippocampus and plays critical roles in memory and synapse formation, as well as exerting antidepressant-like effects in psychiatric disorders. We previously reported that BDNF is expressed in salivary glands and affects blood BDNF content. However, the function of salivary BDNF remains unclear. The aim of this study was to generate transgenic mice overexpressing BDNF in the salivary glands. Hence, we used the Lama construct (hemagglutinin (HA)-tagged mouse *Bdnf* cDNA) to specifically express BDNF in mouse salivary glands. Compared with control mice, *Bdnf*-HA transgenic mice showed increased blood BDNF and expressed salivary BDNF-HA. Molecular analysis revealed enhanced hippocampal BDNF levels and activation of the BDNF receptor, tyrosine kinase B (TrkB), in transgenic mice. In both the open field and elevated-plus maze tests, transgenic mice showed anxiolytic-like behavioral effects compared with control or sialoadenectomized mice. Among downstream components of the BDNF-TrkB signaling pathway, metabolic activation of the γ-aminobutyric acid (GABA) synthetic pathway was found, including higher levels of the GABA synthetic enzyme, glutamate decarboxylase 1 (GAD1). Thus, we have established a transgenic mouse expressing BDNF in the parotid gland that may be useful to examine the hippocampal effects of salivary BDNF.

## 1. Introduction

Brain-derived neurotrophic factor (BDNF) is a neurotrophin that is highly expressed in the hippocampus, and which plays critical roles in memory and synapse formation [1,2]. BDNF is also a major regulator of inhibitory γ-aminobutyric acid (GABA)-ergic neurotransmission [3]. Elevating hippocampal BDNF levels attenuates chronic stress in a mouse model of depression [4], while chronic administration of antidepressant drugs increases BDNF expression within the hippocampus [5,6]. Interestingly, blood and hippocampal BDNF levels are reduced in patients with depression [7,8]. Furthermore, expression of the BDNF receptor, tyrosine kinase B (TrkB), is downregulated in the hippocampus during schizophrenia and mood disorders [9]. Downregulation of hippocampal BDNF-TrkB signaling in schizophrenia and mood disorders is accompanied by changes in glutamate decarboxylase 1 (GAD1) [9], an enzyme involved in production of GABA from glutamate [10]. The link between BDNF-TrkB signaling and GAD1-GABA signaling plays an essential role in improvement of the anxiety state [11]. Although the functional significance of BDNF in the central nervous system is progressing, the role of BDNF in the periphery remains unclear.

Administration of peripheral growth factors exerts anxiolytic-like effects [12,13]. BDNF and TrkB overexpression in the hippocampus of mice under stressed conditions have antidepressant behavioral effects [14,15]. Administration of peripheral BDNF in mice elevates hippocampal BDNF levels and increases the number of entries into open arms of the elevated-plus maze (EPM) [4]. In comparison, mice with chronic stress-induced depression exhibit decreased hippocampal BDNF expression and reduced number of entries into the center area of the open field (OF) test [16]. Improvement of the depressive state by antidepressant drugs is associated with higher blood BDNF levels [7], and blood BDNF levels are positively correlated with salivary BDNF levels under stressful conditions [17]. The salivary glands display both exocrine and endocrine functions [18], and growth factors secreted by the salivary glands may have hormone-like effects on the adrenal gland [19], stomach [20], and heart [21]. Transfer of factors produced by the salivary glands into the bloodstream [22] may exert systemic effects on various organs [17].

Absence of blood BDNF was observed in mice [23]. Furthermore, *Bdnf* expression was not detected by mRNA analysis in salivary glands [24]. Therefore, to determine the role of salivary gland BDNF, we generated transgenic (TG) mice expressing BDNF in the salivary glands. Consequently, in this study, we have performed metabolome analysis (focusing on the hippocampus) and behavioral analysis to determine characteristics of salivary gland-derived BDNF overexpression in TG mice.

## 2. Results

### 2.1. Expression of Brain-Derived Neurotrophic Factor-Hemagglutinin (Bdnf-HA) Transgene

**Figure 1:**
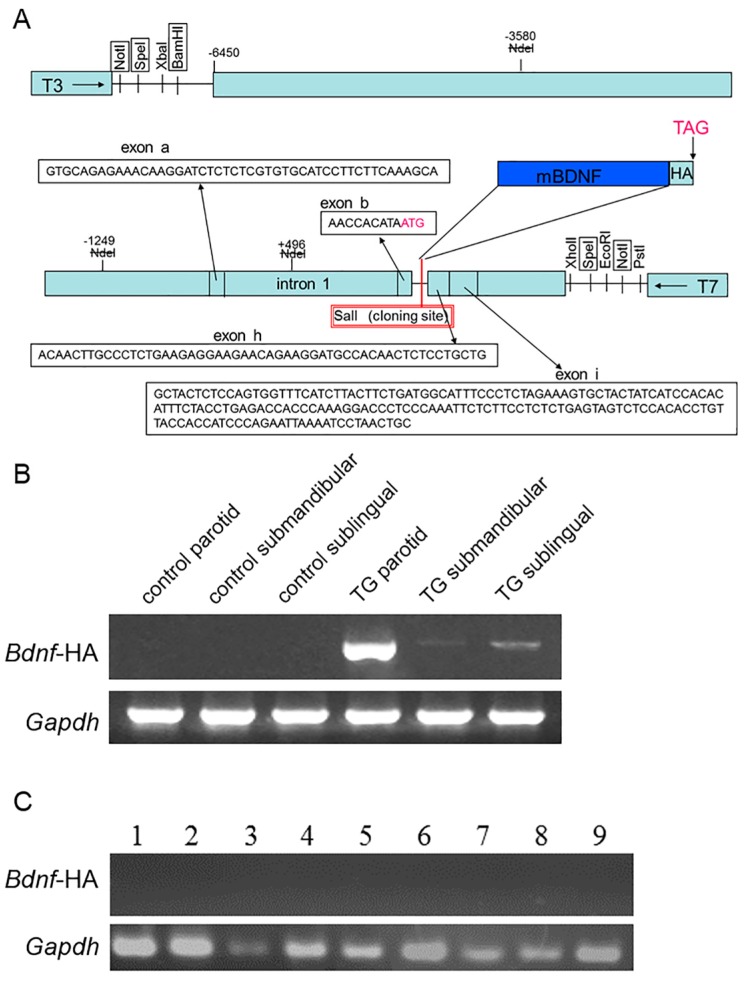
Transgene construct and expression. (**A**) To direct brain-derived neurotrophic factor (BDNF) expression to the salivary glands, mouse *Bdnf* cDNA was linked to the mouse parotid-specific protein promoter. In addition, the construct was linked to a hemagglutinin (HA) tag to distinguish it from endogenous BDNF; (**B**) Transgenic mice (TG) showed markedly elevated *Bdnf*-HA mRNA expression in the parotid gland compared with C57BL/6 control mice; (**C**) In generated transgenic (TG) mice, a number of tissues (excluding the salivary glands) do not express the *Bdnf*-HA gene. 1: cerebral cortex; 2: hippocampus; 3: pituitary gland; 4: spleen; 5: liver; 6: adrenal gland; 7: lung; 8: kidney; 9: heart. Mouse glyceraldehyde-3-phosphate dehydrogenase (*Gapdh*) gene expression was observed in all samples.

We constructed TG lines on a C57BL/6 background, in which expression of mouse *Bdnf* cDNA is regulated by the parotid-specific protein (PSP) promoter (Figure 1A) [25,26]. Of 17 progeny screened by RT-PCR of tail DNA, two (TRM-002 and TRM-014) were positive for the PSP promoter *Bdnf*-hemagglutinin (HA) transgene. TRM-002 has weak fertility. Therefore, in this study, we mainly analyzed *Bdnf*-HA TG mice derived from the TRM-014 mouse. Expression of the *Bdnf*-HA transgene was detected in the salivary glands of *Bdnf*-HA TG mice (Figure 1B). In particular, an intense *Bdnf*-HA specific band was detected in the parotid gland. In contrast, no *Bdnf*-HA transgene expression was noted in C57BL/6 control mice (Figure 1B). Mouse glyceraldehyde-3-phosphate dehydrogenase (*Gapdh*) gene expression was observed in all samples (Figure 1B). The *Bdnf*-HA transgene was not expressed by RT-PCR in nine tissues, namely cerebral cortex, hippocampus, pituitary gland, spleen, liver, adrenal gland, lung, kidney, and heart (Figure 1C).

### 2.2. Analysis of BDNF-HA Protein

**Figure 2:**
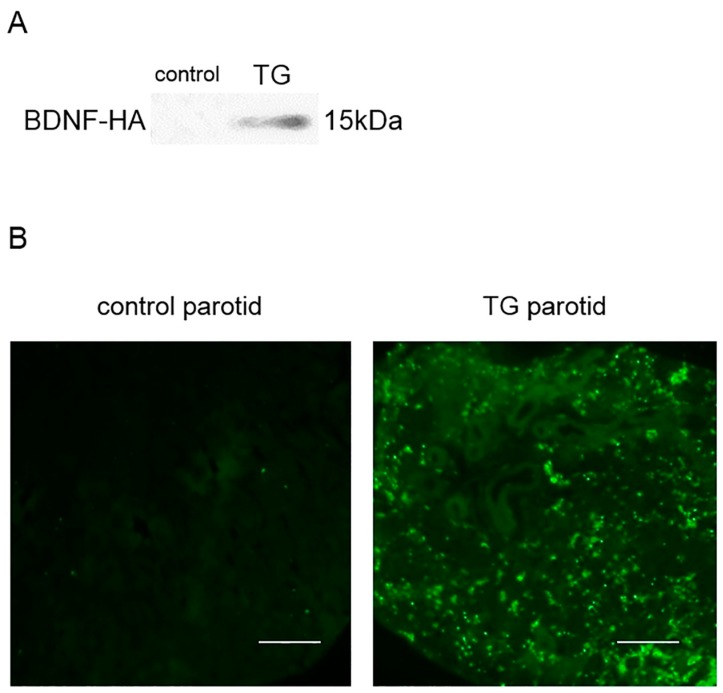
Expression of transgene product. (**A**) BDNF-HA was detected in the saliva of transgenic (TG), but not C57BL/6 control mice; (**B**) Hemagglutinin (HA) immunohistochemistry in the parotid gland. In C57BL/6 control mice, HA protein was not detected. In TG mice, HA protein was detected in acinar cells, but not detected in various duct-type cells or myoepithelial cells. Green color: immuno-positive HA staining. Bar = 100 μm.

In saliva samples, BDNF-HA protein was detected in TG mice, but not C57BL/6 control mice (Figure 2A). BDNF-HA protein was not expressed in nine tissues (Figure S1).

Parotid gland tissue from C57BL/6 control mice revealed no expression of BDNF-HA protein. Although no apparent HA expression was observed in various duct-type cells or myoepithelial cells, intense HA expression was localized in acinar cells in parotid gland tissue of *Bdnf*-HA TG mice (Figure 2B).

### 2.3. Analysis of Endogenous or Total BDNF and Phospho-TrkB

**Figure 3:**
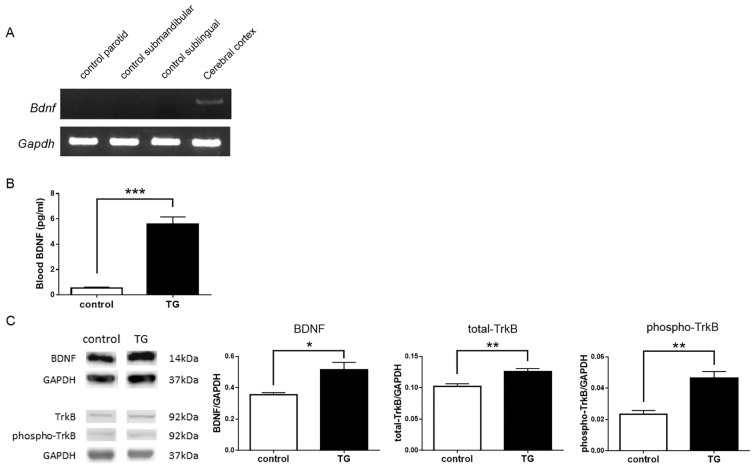
Expression of naive BDNF and phosphorylation of tyrosine kinase B (TrkB) in the hippocampus. (**A**) Mouse *Bdnf* mRNA was not expressed in the salivary glands of C57BL/6 control mice, but was detected in the cerebral cortex. Mouse *Gapdh* gene expression was observed in all samples; (**B**) Blood BDNF levels were significantly higher in transgenic (TG) mice compared with C57BL/6 control mice. Each bar represents mean ± standard error of the mean (SEM) of C57BL/6 control mice (*n* = 4) or TG mice (*n* = 4); *** *p* < 0.0001, Student’s *t*-test; (**C**) Western blots for examining BDNF-TrkB signaling pathway activation in the hippocampus of C57BL/6 control and TG mice. Quantification of immunoblots. Bars represent BDNF/GAPDH and TrkB/GAPDH ratios in C57BL/6 control and TG mice. BDNF, total TrkB, and phospho-TrkB protein levels in the hippocampus were significantly different between C57BL/6 control and TG mice. Each bar represents mean ± SEM of C57BL/6 control mice (*n* = 4) or TG mice (*n* = 5 or 6); * *p* < 0.05, ** *p* < 0.01, Student’s *t*-test.

Endogenous mouse *Bdnf* mRNA was not detected in the salivary glands of C57BL/6 control mice. Mouse *Gapdh* gene expression was observed in all samples (Figure 3A).

Blood levels of BDNF protein were significantly increased in *Bdnf*-HA TG mice (5.584 ± 0.570) compared with C57BL/6 control mice (0.553 ± 0.065; *t* = 8.78, *df* = 6, *p* < 0.0001; Figure 3B).

BDNF levels in the hippocampus were significantly higher in *Bdnf*-HA TG mice (0.514 ± 0.005) compared with C57BL/6 control mice (0.354 ± 0.015; *t* = 2.86, *df* = 7, *p* < 0.05; Figure 3C). Moreover, TrkB expression was significantly elevated in *Bdnf*-HA TG mice (0.126 ± 0.005) compared with C57BL/6 control mice (0.102 ± 0.004; *t* = 3.50, *df* = 8, *p* < 0.05; Figure 3C). Similarly, phospho-TrkB expression was higher in *Bdnf*-HA TG mice (0.047 ± 0.004; control, 0.023 ± 0.003; *t* = 4.08, *df* = 8, *p* < 0.05; Figure 3C).

### 2.4. Anxiolytic-Like Behavior in Bdnf-HA Generated Transgenic (TG) Mice

**Figure 4:**
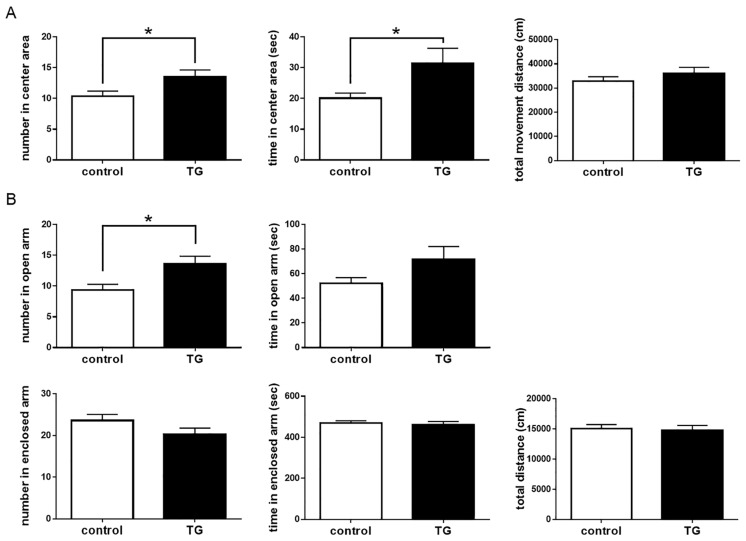
Behavioral performance in transgenic (TG) mice. (**A**) In the open field (OF) test, total number of entries in the center of the OF was significantly different between C57BL/6 control and TG mice. Each bar represents mean ± SEM of C57BL/6 control mice (*n* = 13) or TG mice (*n* = 13); * *p* < 0.05, Student’s *t*-test; (**B**) In the elevated-plus maze (EPM) test, total number of entries and total time in open arms were significantly different between the two groups of mice. Each bar represents mean ± SEM of C57BL/6 control mice (*n* = 13) or TG mice (*n* = 13); * *p* < 0.05, Student’s *t*-test.

To assess the impact of salivary BDNF on behavioral action, we investigated differences between both mice using the OF and EPM tests. Number of entries and time spent in the center of the OF were significantly different between C57BL/6 control and *Bdnf*-HA TG mice (number: *t* = 2.29, *df* = 24; time: *t* = 2.15, *df* = 24; *p* < 0.05), but there was no difference in total distance traveled between these two groups (*p* = 0.33; Figure 4A). In the EPM, *Bdnf*-HA TG mice showed a significantly higher number of open arm entries compared with C57BL/6 control mice (*t* = 2.76, *df* = 24, *p* < 0.05). The time spent in open arms was longer in *Bdnf*-HA TG mice than C57BL/6 control mice (*p* = 0.11), while number of enclosed arm entries was lower in *Bdnf*-HA TG mice compared with C57BL/6 control mice (*p* = 0.12). There were no differences between the groups in time spent in enclosed arms (*p* = 0.60) or total distance (*p* = 0.84; Figure 4B). These results indicate an anxiolytic-like effect of salivary BDNF in *Bdnf*-HA TG mice.

### 2.5. Anxiolytic-Like Behavior of Sialoadenectomized Bdnf-HA TG Mice

**Figure 5:**
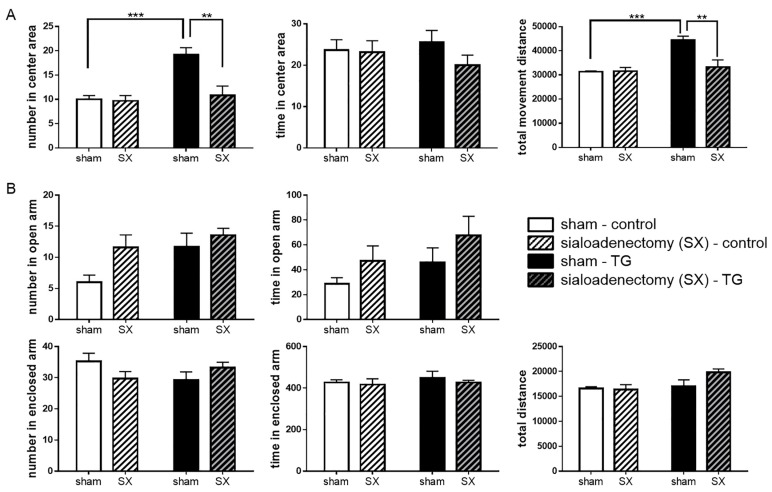
Behavioral effect of sialoadenectomy. (**A**) Total number of entries into the center of the open field (OF) was significantly decreased in sialoadenectomized transgenic (TG) mice. Each bar represents mean ± SEM of C57BL/6 control mice (*n* = 7) or TG mice (*n* = 7); ** *p* < 0.01, *** *p* < 0.001, two-way ANOVA followed by Tukey’s test; (**B**) In the elevated-plus maze (EPM), total number of entries and total time spent in the various arms were not significantly different between the two groups of sialoadenectomized mice. Each bar represents mean ± SEM of C57BL/6 control mice (*n* = 7) or TG mice (*n* = 7).

Sialoadenectomized *Bdnf*-HA TG mice showed a significant decrease in number of entries into the center area of the OF test compared with sham-operated *Bdnf*-HA TG mice, with the number of entries comparable to sham-operated C57BL/6 control mice (*p* < 0.01; Figure 5A). Sialoadenectomized C57BL/6 control mice showed no change in the OF test compared with sham-operated C57BL/6 control mice, although total distance traveled was significantly different between the two groups of sham-operated mice. In the EPM, there were no significant differences between the various groups (Figure 5B). 

### 2.6. Metabolome Analysis in the Hippocampus

**Figure 6:**
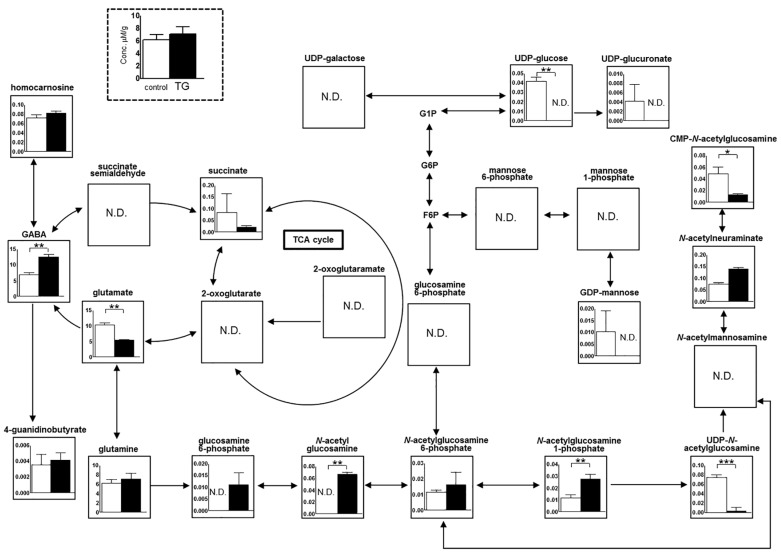
Metabolic analyses of the hippocampus. Metabolomic profiles in the hippocampus were analyzed by capillary electrophoresis-time of flight-mass spectrometry. Several metabolites, including amino acids, were found at higher levels in the hippocampus of *Bdnf*-HA transgenic (TG) mice compared with C57BL/6 control mice. Each bar represents mean ± SEM of C57BL/6 control mice (*n* = 3) or TG mice (*n* = 3); N.D. indicated not detected. * *p* < 0.05, ** *p* < 0.01, *** *p* < 0.001, Student’s *t*-test. One way arrow; direct conversion between metabolites, bidirectional arrow; inter-changeable metabolites.

The key metabolic pathways in the hippocampus are schematically shown in Figure 6. Several metabolites, including amino acids, were found at higher levels in the hippocampus of *Bdnf*-HA TG mice compared with C57BL/6 control mice, although the differences were not significant. In both groups of mice, metabolic components of GABA and glutamate showed inverse correlations. However, *Bdnf*-HA TG mice exhibited significantly higher levels of GABA, *N*-acetyl glucosamine, and *N*-acetylglucosamine 1-phosphate (*p* < 0.01), while C57BL/6 control mice displayed higher levels of glutamate, uridine diphosphate (UDP)-glucose, cytidine monophosphate-*N*-acetylglucosamine (*p* < 0.01), and UDP-*N*-acetylglucosamine (*p* < 0.001). The consistent increasing of *N*-acetyl glucosamine, *N*-acetylglucosamine 1-phosphate, and UDP-*N*-acetylglucosamine indicated the overall enrichment of downstream metabolite of glutamate.

### 2.7. Glutamate Decarboxylase 1 (Gad1) mRNA Expression in the Hippocampus

**Figure 7:**
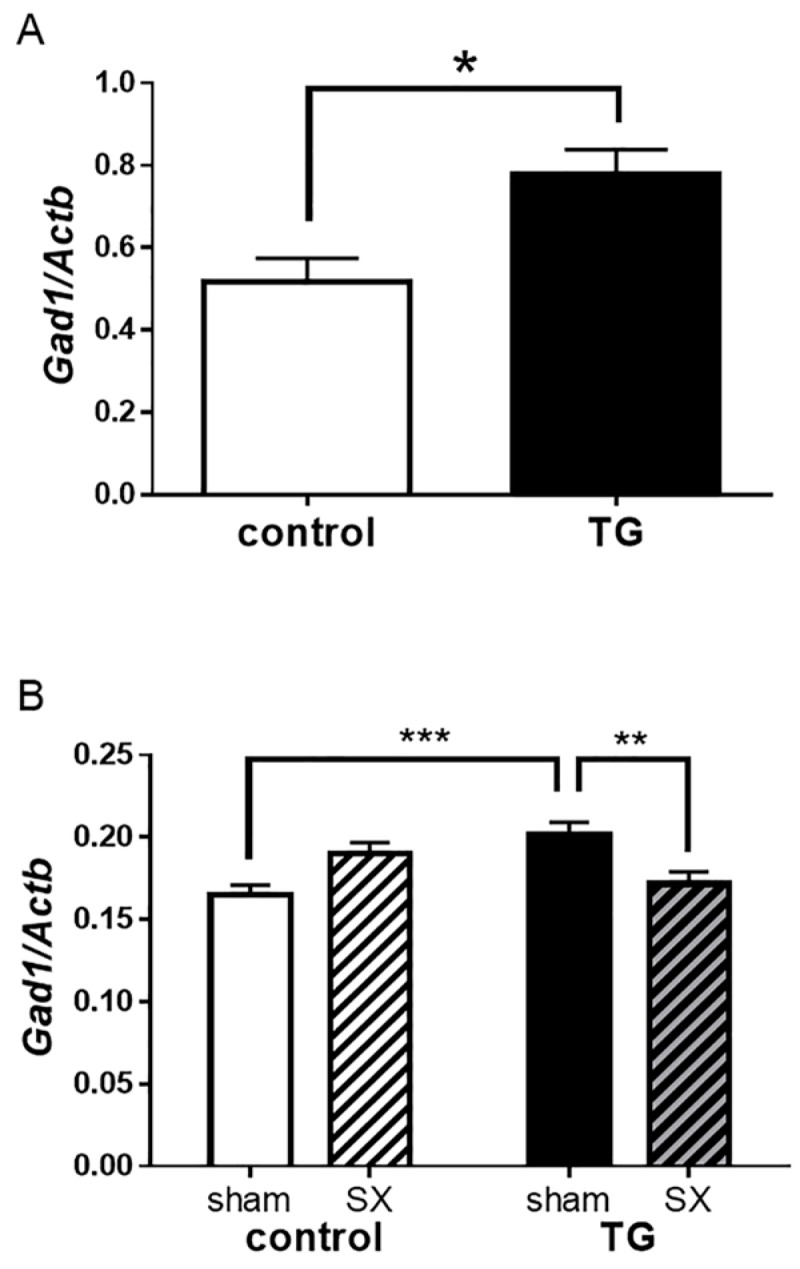
Expression of *Gad1* in the hippocampus. (**A**) Glutamate decarboxylase 1 (*Gad1*) mRNA levels were quantified by real-time PCR and normalized to β-actin (*Actb*) mRNA levels. In transgenic (TG) mice, *Gad1* expression levels were significantly increased compared with C57BL/6 control mice. Each bar represents mean ± SEM of C57BL/6 control mice (*n* = 6) or TG mice (*n* = 6); * *p* < 0.05, Student’s *t*-test; (**B**) Expression of *Gad1* was significantly decreased in sialoadenectomized TG mice to similar levels as C57BL/6 control mice. Each bar represents mean ± SEM of C57BL/6 control mice (*n* = 6) or TG mice (*n* = 6); ** *p* < 0.01, *** *p* < 0.001, two-way ANOVA followed by Tukey’s test. sham: sham-operated mice; SX: sialoadenectomized mice.

Subsequently, we investigated the impact of salivary BDNF on GABA pathways in the hippocampus, focusing on GABA-associated factors. *Gad1* mRNA expression in the hippocampus was higher in *Bdnf*-HA TG mice (0.777 ± 0.062) compared with C57BL/6 control mice (0.518 ± 0.058; *t* = 3.13, *df* = 10, *p* < 0.05; Figure 7A).

To further clarify the effect of salivary BDNF in the hippocampus, we analyzed *Gad1* mRNA levels in the hippocampus of sialoadenectomized transgenic mice. *Gad1* expression was significantly reduced in sialoadenectomized transgenic mice compared with sham-operated transgenic mice (*p* < 0.01; Figure 7B).

## 3. Discussion

Characteristics of *Bdnf*-HA TG mice are shown in Section 2.1, Section 2.2 and Section 2.3. These results show that the *Bdnf*-HA transgene was successfully introduced into C57BL/6 mice and expressed in the salivary glands, especially the parotid gland. Moreover, this suggests that the PSP promoter is suitable for expressing substances in the salivary gland. In addition, since BDNF expression was not observed in the salivary glands of C57BL/6 mice, an advantage of these TG mice is that there is no influence of endogenous BDNF from the salivary glands. Furthermore, immunohistochemical analysis detected BDNF-HA immunopositivity in acinar cells, confirming that salivary BDNF-HA protein is produced in the salivary glands. *Bdnf*-HA transgene expression also increased the amount of blood BDNF. Although only a trace amount was detected as BDNF content, this was a 10-fold increase, which is a marked increase. BDNF-HA protein is not expressed in various organs, but is observed in the salivary glands. We have reported that the salivary glands are a likely organ from which blood BDNF originates [17]. Although the transition path of BDNF-HA protein from the salivary glands to blood is unknown, *Bdnf*-HA TG mice are characterized as mice with high levels of blood BDNF content, in contrast to C57BL/6 mice.

*Bdnf*-HA TG mice show that increased BDNF levels in the hippocampus have an anxiolytic-like effect in the OF and EPM tests. This is consistent with previous studies in which BDNF was administered into the hippocampus [27], midbrain [28], or peripherally [4], as well as a report in which BDNF was overexpressed in forebrain structures of TG mice [14]. Hippocampal and peripheral BDNF has an anxiolytic-like behavioral effect in animal models of anxiety and depression [4,14,29]. These observations are supported by our data showing activation of TrkB signaling in the hippocampus of TG mice. TrkB overexpression in the hippocampus reduces anxiety-like behavior in response to stress vulnerability [30]. Increased BDNF expression is accompanied by upregulation of TrkB expression [5]. Similar to overexpression of TrkB [15], increased hippocampal BDNF produced by peripheral administration of neurotrophin has antidepressant effects [14,28]. Since the amount of blood BDNF increases in *Bdnf*-HA TG mice, this suggests that the anxiolytic behavior of these mice may be influenced.

We examined levels of GABA-related metabolites in TG animals using metabolomic analysis. Metabolomic profiles for the GABA and glutamate systems differ strikingly between TG and wild-type mice, with TG animals showing an increase in GABA release. This is consistent with previous studies reporting that BDNF in hippocampal neurons enhances GABA synthesis [31]. Interestingly, TG mice exhibit upregulation of *Gad1* expression in the hippocampus. BDNF-TrkB signaling is closely related to *Gad1* expression in depression and anxiety [11,32], and regulates GABAergic mechanisms by inducing *Gad1* expression [32]. To assess the effect of salivary BDNF on GABAergic neurotransmission, we investigated *Gad1* mRNA expression in sialoadenectomized mice. Expression levels were decreased in sialoadenectomized TG mice compared with sham-operated TG mice. Indeed, levels were decreased to similar levels as sialoadenectomized and non-sialoadenectomized C57BL/6 control mice.

Various outcomes of BDNF overexpression in mice have been reported. Croll et al. reported that BDNF overexpression in the brain has deleterious effects on the central nervous system [33]. BDNF overexpressing mice exhibit learning deficits and memory impairments [34]. However, in these mice, BDNF expression was at considerably high levels (3.3-fold vs. control) [34]. In comparison, in our study, mice show a smaller change in expression levels (1.5-fold vs. control), although the difference was still significant. Nonetheless, the change in hippocampal BDNF levels in our study is similar to a previous report in which hippocampal BDNF levels were increased by peripheral BDNF administration [4]. Altogether, these phenomena indicate that BDNF expression in the salivary glands may affect hippocampal metabolism.

## 4. Materials and Methods

### 4.1. Generation of Transgenic Mice

Design of the transgene used the Lama vector [35]. The Lama construct (HA-tagged mouse *Bdnf* cDNA) was expressed in mouse salivary glands by endogenous PSP (Figure 1A). Mouse *Bdnf* cDNA was cloned into the pCI vector (Promega Corp., Madison, WI, USA). The expression vector was confirmed by detection of the fusion protein before transfection. The fragments containing mouse *Bdnf* cDNA were microinjected into the fertilized eggs, which obtain from C57BL/6 mice, by previously reported method [25,26]. Next, TG mice were backcrossed onto a C57BL6 background. When the mice were 4 weeks of age, DNA was extracted from tails using the Qiagen DNeasy Tissue kit (Qiagen Inc., Valencia, CA, USA), and PCR performed using primers specific for the *Bdnf* construct (forward primer: 5′-GCT CTA GAA TGT TCC ACC AGG TGA GAA-3′; reverse primer: 5′-AGC GTA ATC TGG AAC ATC GTA TGG GTA-3′) to identify TG mice. In TG mice, PCR products contain the mutant band, while PCR products in C57BL/6 control mice have no band.

In all experiments, 7–9-week-old male mice were used. Mice were housed in groups of three to six per cage, and had free access to food and water. Mice were maintained under pathogen-free and temperature- and humidity-controlled conditions (22 ± 3 °C, 55 ± 2%), with a light/dark cycle of 12 h (lights off at 7:00 p.m.). The experimental protocol used in this study was reviewed and approved by the Ethics Committee for Animal Experiments of Kanagawa Dental University (approval number 20100212-223, project date: 12 February 2010) and was performed in accordance with the Guidelines for Animal Experimentation of Kanagawa Dental University and the ARRIVE guidelines for reporting animal research.

### 4.2. Sialoadenectomy Model

Transgenic mice, 5–7 weeks old, had their parotid glands resected in accordance with a well-established protocol, and were maintained under pathogen-free conditions for 2 weeks [36,37]. Under sodium pentobarbital anesthesia (50 mg/kg, intraperitoneally (i.p.)), a 2-cm skin incision was made in the neck, and the parotid gland removed bilaterally. Mice were placed on a hotplate after the operation. To confirm resection of parotid tissue, hematoxylin and eosin staining was performed using formalin-fixed paraffin-embedded tissue. Control mice underwent a sham operation. Mice were randomly assigned to these operations (six to seven mice per experimental group). These mice (7–9 weeks old) were also used for assessment of *Gad1* mRNA expression and behavioral analyses.

### 4.3. Sample Collection

Mice were anesthetized with sodium pentobarbital (50 mg/kg, i.p.) for collection of saliva, blood, and tissue samples. For saliva samples, mice were injected with pilocarpine-HCl (1 mg/kg, i.p.; Sanpilo 1%; Santen Pharmaceutical Co., Ltd., Osaka, Japan) under anesthesia. Saliva samples were collected from the oral cavity using capillary pipettes (ringcaps; Hirschmann Laborgerate GmbH & Co., KG, Eberstadt, Germany). Collected blood samples were immediately placed on ice, centrifuged at 760× *g* for 15 min at 4 °C, and stored at −80 °C until analyses [38].

### 4.4. RNA Isolation and RT-PCR

Total RNA isolation was performed using the ISOGEN Kit (Nippon Gene, Toyama, Japan) according to the manufacturer’s instructions [39]. RNA concentration was determined with a Bio Spec-nano spectrophotometer (Shimadzu Access Corp., Kanagawa, Japan). Total RNA was reverse transcribed using a first-strand cDNA synthesis kit (Roche Diagnostics Ltd., Lewes, UK) according to the manufacturer’s instructions. RT-PCR was performed using Takara Ex-Taq^™^ (Takara Bio., Shiga, Japan) according to the manufacturer’s instructions. *Bdnf*-HA-specific primers were: 5′-AGC GTA ATC TGG AAC ATC GTA TGG GTA-3′ (forward) and 5′-GCT CTA GAA TGT TCC ACC AGG TGA GAA-3′ (reverse). PCR cycling conditions were: 25 cycles of 95 °C for 30 s, 60 °C for 30 s, and 72 °C for 1 min for *Bdnf*-HA. Glyceraldehyde-3-phosphate dehydrogenase (*Gapdh*) was used as the housekeeping gene.

### 4.5. Immunoblotting

Protein from whole saliva and hippocampal lysates were resolved by gel electrophoresis [40]. The protein migration during electrophoresis were monitor using Precision Plus All Blue Standard (Bio-Rad, Tokyo, Japan). Proteins were transferred onto 0.2 μm PVDF membranes (Merck Millipore, Billerica, MA, USA) in transfer buffer (25 mM Tris, 190 mM glycine, 20% MeOH) for 1 h at 15 V, blocked with 5% non-fat dry milk (BDNF, phospho-TrkB) or 3% BSA (TrkB) in PBS (0.1% Tween-20, 1% NP-40), and probed overnight with anti-HA rabbit polyclonal antibody (Ab) (1:1000; C29F4, Cell Signaling Technology, Beverly, MA, USA), anti-BDNF rabbit polyclonal Ab (1:1000; molecular weight 14 kDa; sc-546, Santa Cruz Biotechnology, Santa Cruz, CA, USA), anti-TrkB rabbit polyclonal Ab (1:1000; molecular weight 92 kDa; ab51190, Abcam, Cambridge, MA, USA), anti-phospho-TrkB rabbit polyclonal Ab (1:1000; molecular weight 92 kDa; ab52191, Abcam), or anti-GAPDH rabbit monoclonal Ab (1:1000; molecular weight 37 kDa; 14C10, Cell Signaling Technology). After washing in PBS-Tween (PBST; 0.1% Tween 20, 50 mM Tris, pH 7.6, 150 mM NaCl), membranes were incubated in anti-rabbit IgG conjugated with horseradish peroxidase (HRP) (Dako Cytomation, Glostrup, Denmark), diluted 1:2000 in blocking buffer for 1 h at room temperature. Membranes were washed again and Luminata^™^ Forte Western HRP Substrate (Merck Millipore) used for detection. The ChemiDoc XRS system (Bio-Rad) was used to scan blots. Bands were analyzed using Quantity One 1-D analysis software (version 4.6., Bio-Rad).

### 4.6. Immunohistochemistry

Immunohistochemistry was performed using 8-μm sections with fixed in cold acetone for 5 min. The sections were dried in cool air for 30 min, and were washed in phosphate buffered saline (PBS, 0.01 M, pH 7.4).After this, they were blocked in 10% rabbit serum for 30 min at room temperature. The primary antibody, anti-HA rabbit polyclonal Ab (1:1600; C29F4; Cell Signaling Technology), was applied for 1 h at room temperature. After three washes with PBS (5 min each), sections were incubated in PBS with polyclonal swine anti-rabbit immunoglobulins/FITC (Dako Denmark A/S., Glostrup, Denmark) at a dilution of 1:100 for 1 h at room temperature [41,42]. Immunofluorescent signals were detected using Biozero BZ-8000 (Keyence Co., Osaka, Japan).

### 4.7. ELISA Analysis

Blood BDNF was quantified by sandwich enzyme-linked immunosorbent assay (ELISA) (BDNF Emax Immunoassay System; Promega Corp.), according to the manufacturer’s instructions [36,37]. All assays were performed in Costar 96-well ELISA plates (Corning Inc., Acton, MA, USA). After conjugate of HRP and color developed, each sample measured at a wavelength of 450/570 nm. The content was quantified by a standard curve generated using known amounts of BDNF, and with a detection sensitivity <7.8 pg/mL.

### 4.8. Behavioral Tests

The OF and EPM tests were used to assess anxiety-like behavior in mice [4]. The test apparatus was surrounded by a black curtain and illuminated with a fluorescent light (light density: 20–25 l×) [43]. At least 1 h before the beginning of the first trial of the day, mice were brought to the experimental room. An increase in total time spent or number of entries into the center of the open field in the OF test or into open arms in the EPM test was considered to represent an anxiolytic effect. Activity was videotaped and measured using TopScan (Clever Sys Inc., Reston, VA, USA). All behavioral tests were performed between 3 and 7 p.m. and instruments were cleaned after each trial with 30% (*v*/*v*) ethanol solution. In the OF test, each mouse was placed into the center area of the OF (90 × 45 cm) and allowed to explore for 5 min [44]. Parameters assessed were distance moved, time, and entries into the center. At least two days after the end of the OF test, each mouse was tested in the EPM. A mouse was placed in the center of the EPM, facing one of the open arms [4], and tested for 10 min. Total number of entries, time spent in each arm, and total distance moved were recorded. Entries were defined when the center of the body crossed into a particular arm. The EPM test consists of a plus-shaped maze with two open arms (30 × 5 cm) and two enclosed arms (30 × 5 × 20 cm), spreading out from a central platform (5 × 5 cm), with the maze elevated at a height of 50 cm from the floor.

### 4.9. Metabolic Analyses of the Hippocampus

#### 4.9.1. Metabolomic Analyses

Metabolomic analyses were performed as described elsewhere [45,46] with slight modifications.

#### 4.9.2. Processing of Tissue

Frozen tissue (approximately 50 mg) was immediately plunged into methanol (500 μL) and 2-(*N*-morpholino) ethanesulfonic acid. Samples were then homogenized at 1500 rpm for 5 min using a Shake Master Neo (BMS, Tokyo, Japan) to inactivate enzymes. Next, 200 μL Milli-Q water and 500 μL chloroform were added, and the solution centrifuged at 4600× *g* for 5 min at 4 °C. The upper aqueous layer was centrifugally filtered at 9100× *g* for 3.5 h at 4 °C through Millipore 5-kDa cutoff filters to remove large molecules. The filtrate (300 μL) was lyophilized and dissolved in 50 μL Milli-Q water containing a reference compound (200 μM of 3-aminopyrrolidine and trimesate) prior to capillary electrophoresis-time of flight-mass spectrometry analysis. The measurement condition and instrument parameters for cation and anion profiles were described elsewhere [47].

#### 4.9.3. Data Analysis for Metabolomic Data

Raw data were analyzed using MasterHands [48,49] to produce the concentration matrix. The concentration of each metabolite was calculated using a ratio of internal standards and standard mixture. Data were visualized as bar graphs (average and standard deviation) in GraphPad Prism (v. 5.0.; GraphPad Software Inc., San Diego, CA, USA), and assigned on manually curated metabolic pathways.

### 4.10. Real-Time PCR

RNA isolation and cDNA synthesis were performed as described above. Real-time PCR was performed using the LightCycler system (Roche Diagnostics Ltd.) according to the manufacturer’s instructions [42]. The primer sequences used to amplify *Gad1* were 5′-TCC TGG TTG ACT GTA GAG ACA C-3′ (forward) and 5′-CAT ATT GGT ATT GGC AGT CGA T-3′ (reverse). Primers were designed and synthesized by Nihon Gene Research Laboratory (Sendai, Miyagi, Japan). The thermocycling parameters for *Gad1* were: 95 °C for 10 min, followed by 35 cycles of 95 °C for 10 s, 62 °C for 10 s, and 72 °C for 10 s. Mouse β-actin (*Actb*) was used as the housekeeping gene. Gene expression is given as the ratio of *Gad1* transcript number to *Actb* transcript number.

### 4.11. Statistical Analysis

Statistical analyses were performed using GraphPad Prism (v. 6.05.; GraphPad Software Inc.). Values are reported as mean ± SEM. Data were analyzed using Student’s *t*-test or two-way (transfection × sialoadenectomy) analysis of variance (ANOVA) followed by Tukey’s test. *p* values < 0.05 were considered statistically significant.

## 5. Conclusions

In this study, TG mice expressing BDNF in the salivary gland were prepared and characterized. Consequently, the mice characteristically show increased blood BDNF and anxiolytic behavior. These TG mice may be useful to investigate the salivary gland-brain relationship. However, the problem in this study is that salivary gland-derived BDNF does not provide direct evidence for induction of TrkB phosphorylation in the hippocampus. It is now necessary to analyze the function of salivary gland-derived BDNF using this mouse.

## Supplementary Materials

Supplementary materials can be found at www.mdpi.com/1422-0067/18/9/1902/s1.

## Abbreviations

Ab	Antibody
ANOVA	Analysis of variance
BDNF	Brain-derived neurotrophic factor
ELISA	Enzyme-linked immunosorbent assay
GABA	γ-Aminobutyric acid
GAD1	Glutamate decarboxylase 1
GAPDH	Glyceraldehyde-3-phosphate dehydrogenase
HA	Hemagglutinin
HRP	Horseradish peroxidase
OF	Open field
SEM	Standard error of the mean
TG	Transgenic
TrkB	Tyrosine kinase B
UDP	Uridine diphosphate
