# Supplementary material for: Salivary Gland Derived BDNF Overexpression in Mice Exerts an Anxiolytic Effect
Source: Int J Mol Sci. 2017 Sep 5;18(9):1902. doi: 10.3390/ijms18091902 (PMC5618551; doi:10.3390/ijms18091902)
Supplement: Supplementary file 1 [file ijms-18-01902-s001.pdf]

Supplementary Materials: Salivary Gland Derived BDNF Overexpression in Mice Exerts an Anxiolytic Effect

Juri Saruta [1,†], Masahiro To [1,†], Masahiro Sugimoto [1,2,3], Yuko Yamamoto [4], Tomoko Shimizu [5], Yusuke Nakagawa [1], Hiroko Inoue [6,7], Ichiro Saito [6] and Keiichi Tsukinoki [1,*]

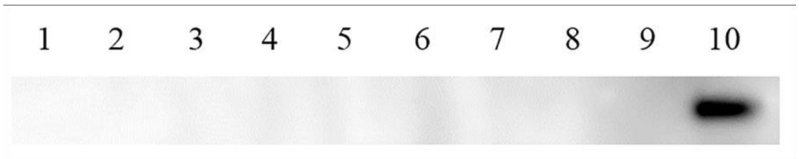

Figure S1. BDNF-HA protein expression in transgenic mice. In transgenic (TG)mice, a variety of organs,excluding the salivary glands, donot expressthe BDNF-HAprotein(1: cerebral cortex;2: hippocampus;3: pituitary gland;4: spleen;5: liver;6: adrenal gland;7: lung;8: kidney;9: heart;10: positivecontrol).
